# Application of Pineapple Leaves as Adsorbents for Removal of Rose Bengal from Wastewater: Process Optimization Operating Face-Centered Central Composite Design (FCCCD)

**DOI:** 10.3390/molecules25163752

**Published:** 2020-08-18

**Authors:** Siham S. Hassan, Ahmed S. El-Shafie, Nourhan Zaher, Marwa El-Azazy

**Affiliations:** Department of Chemistry and Earth Sciences, College of Arts and Sciences, Qatar University, Doha 2713, Qatar; s.hersi@qu.edu.qa (S.S.H.); aelshafie@qu.edu.qa (A.S.E.-S.); nm1601246@qu.edu.qa (N.Z.)

**Keywords:** green adsorbents, pineapple leaves, rose bengal (RB) dye, face-centered central composite design (FCCCD), percentage removal (%R), adsorption capacity (*q_e_*)

## Abstract

Adsorptive removal of rose bengal (RB) from contaminated water samples was approached using pineapple leaves (PAL). Three adsorbents were utilized for that purpose; raw pineapple leaves (RPAL) and the thermally activated bio-waste leaves at 250 and 500 °C. Two measures were executed to evaluate the functionality of exploited biomasses; percentage removal (%R) and adsorption capacity ($q_{e}$). Face-centered central composite design (FCCCD) was conducted to experiment the influence of variables on the %R. Dose of PAL as adsorbent (AD), concentration of RB (DC), pH and contact time (CT), were the inspected factors. Existence of functional groups and formation of activated carbon was instigated employing Fourier-transform infrared (FT-IR) and Raman spectroscopies. Scanning electron microscopy (SEM) and energy-dispersive X-ray spectroscopy (EDX) analyses were used to explore surface features. Thermal behavior of adsorbents was studied using thermogravimetric analysis (TGA). The surface area and other surface structural properties were established using the Brunauer Emmett-Teller (BET) analysis. An amount of 92.53% of RB could be removed with an adsorption capacity of 58.8 mg/g using a combination of pH 5.00 ± 0.20, RPAL dose of 0.05 mg/50 mL, and 10-ppm RB for 180 min. Equilibrium studies divulge a favorable adsorption that follows the Freundlich isotherm. Pseudo-second-order model explains the observed adsorption kinetics.

## 1. Introduction

Water is perceived as the most important renewable source of life, where surface and ground water play major roles in agriculture, livestock production, hydropower generation, etc. The rate of growth of the world population is increasing day after day. This escalating growth is logically associated with several environmental concerns. Water pollution is one of the most serious apprehensions that living creatures have ever faced, if not the most challenging at all. The quality of water is particularly significant for human health. As per the World Health Organization (WHO) reports, poor water quality is responsible for 2.2 million deaths annually. Moreover, more than 2/3 of infant deaths stem from waterborne diseases [1,2,3,4].

Numerous contaminants contribute to water pollution. Among these pollutants, heavy metals, anions (sulfates, phosphates, fluoride, etc.), dyes, pesticides, fertilizers, and pharmaceuticals are the most common [4,5,6,7,8,9,10]. Dyes, the topic of the current investigation, are widely applied in various industries, e.g., paper, cosmetics, paint and textiles production, food processing, etc. Discharge of the industrial effluents into water bodies causes not only a direct mutilation of water physicochemical features (such as color, pH, salinity, organic carbon content, etc.), but also instigates detrimental effects on the ecosystem and consequently the human health. This effect is exacerbated by the diverse chemical structure of these dyes and their resistance to biodegradation [11,12,13].

Rose bengal (RB), a basic xanthene dye, also known as ‘C.I. 45440 and C.I. Acid Red 94′, is chemically recognized as disodium-4,5,6,7-tetrachloro-3′,6′-dihydroxy-2′,4′,5′,7′-tetraiodo-3*H*-spiro [isobenzofuran-1,9′-xanthen]-3-one (molar mass: 1017.64 g/mol). Sodium salt of RB is commonly used in diagnosing eye damage via staining the corneal and conjunctival cells. Other applications of RB include treatment of certain cancers (melanoma and breast cancers), skin conditions such as psoriasis and also as antibacterial. Moreover, RB is extensively used in fabric and photochemical manufacturing. Nonetheless, RB has shown serious impacts on human health, especially when it gets in contact with skin and eyes causing discomfort, irritation, redness and blistering [14,15,16,17,18]. Removal of RB from wastewater has been done implementing various methods such as photo-degradation, nanofiltration and adsorption [19,20,21,22].

Adsorption is one of the most promising strategies for wastewater treatment. On one hand, adsorption is convenient, easy to maneuver, and can be conducted using readily available materials. Conversely, dyes in specific are premeditated to be chemically stable with long–standing photolytical properties. Most of the used strategies for removal of dyes require pre- and post-treatment steps. In addition, majority of these methods are either impractical (request a tedious experimental setup) or expensive with reduced removing capabilities [6,7,8,9,10,11,18,23,24]. Moreover, some of these techniques might not be efficient at low pollutant concentrations. Adsorption is therefore a reasonable choice. Developing the model adsorbent and how the adsorption process is conducted are the keywords in managing the adsorption process. Agricultural as well as industrial wastes represent a real burden on the ecosystem if not appropriately recycled and reprocessed. Sources of agricultural wastes are variable. Yet, by-products of the agricultural processing such as peels, pits, shells, leaves, etc. represent important naturally occurring resources that are copiously available and should be thoroughly thought of for the production of value-added materials [24].

Pineapples (PA, *Ananas comosus*, Family: Bromeliaceae) is a perennial herbaceous plant. PA fruit is mostly planted in coastline and tropical areas. In India, for example, PA fruits are grown on around 2,250,000 acres of land. The first bud of the leaves looks attractive. Later on, leaves become stiff; sword shaped and spirally assembled around the fruit [25,26]. Leaves represent the waste biomass of PA fruits and are commonly used as a source of natural fibers. Amount of waste produced from PA (leaf waste) is worrying, where approximately 20,000–25,000 tons per acre are left out after the harvesting process [27]. Leaf fibers consist of mainly holocellulose and lignin with minor amounts of ash [28]. Raw and activated PAL have shown a promising removal potential for different kinds of contaminants. Table 1 shows an evaluation for the performance of PA through different studies with different adsorbates [29,30,31,32,33,34,35].

As previously mentioned, having an ideal adsorption process could be managed by not only developing the model adsorbent, but also by engineering the adsorption process and more specifically the influencing variables. Different parameters are known to affect the interaction between the adsorbent and the adsorbate such as adsorbent dosage (AD), concentration of the adsorbate (DC), contact time (CT), pH, surface area, as well as the nature of the adsorbent and the pollutant. The conventional strategy for investigating the influence of these variables on the adsorption capability of an adsorbent is to scrutinize the effect of a single variable per time (univariate analysis). This stratagem and in addition for being time and effort consuming, involves several experimentations, an issue that jeopardizes the method greenness. Moreover, this univariate-based strategy does not yield the adequate amount of data that enable the researcher to draw the full picture for the adsorption process. Nevertheless, and since the objective is to build a green bioremediation strategy, coupling of the adsorption process to factorial designs would overcome these concerns [36].

Offering irresistible advantages including saving of time, efforts, and resources, a response surface methodological approach (RSM)–face-centered central composite design (FCCCD) will be utilized in the current approach to optimize the investigated responses. Factorial levels for four independent variables will be adjusted with the target being set to maximize the removal of the studied contaminant (RB) using PAL (raw and thermally treated at 250 and 500 °C, labelled as TTPAL250 and TTPAL500, respectively) as adsorbents. The amount of RB dye adsorbed will be analyzed using spectrophotometry. TGA, FT-IR, SEM, Raman, EDX, CHN, and BET analyses will be used to characterize the prepared adsorbents. To further study the nature of the adsorbents and adsorption process, both kinetic and equilibrium studies will be performed.

## 2. Results and Discussion 

### 2.1. Selection of the Best Performing Adsorbent

Performance of the three prepared adsorbents was measured in terms of %R and the adsorption capacity ($q_{e}$) and using Equations (1) and (2), respectively. Table 2 shows a comparison between the three prepared adsorbents under the same conditions. As per the results revealed in Table 2, RPAL had the highest %R and $q_{e}$ and therefore was further used in the subsequent studies:

(1) $$\left(\%R\right)=\frac{C_{0}-C_{e}}{C_{0}}\times 100\%,$$

(2) $$\left(q_{e}\right)=\frac{C_{0}-C_{e}}{W}V$$

where $C_{0}$ (mg L^−1^) denotes the initial concentration of RB solution, $C_{e}$ is the concentration of the RB solution at equilibrium, V stands to the volume of the solution (L), and W is the weight of the adsorbent used (g).

### 2.2. Response Surface Methodology (RSM): FCCCD

As previously mentioned, the purpose of the current approach is to investigate and optimize the adsorption capability of PAL to RB dye from artificially contaminated water samples. The novelty of the current approach stems from using a multivariate platform that surmounts all the previous cons of the univariate approach. FCCCD, as mentioned, was the design of choice, where the impact of four variables on a single response was assessed and optimized. Central composite designs (CCD) usually contain built-in points from preceding full/fractional designs. In the current case, a full factorial design was the preceding design and the value of alpha (α) or the distance between the axial points and the center was equal to one, denoting a FCCCD [36]. The measured response (%R) was calculated using the formula shown in Equation (1). Conducted experimental runs (as executed by the design setup) as well as the working factorial limits accompanied by the observed and predicted responses are shown in Table 3.

### 2.3. Investigation of Statistically Significant Variables

**Figure 1:**
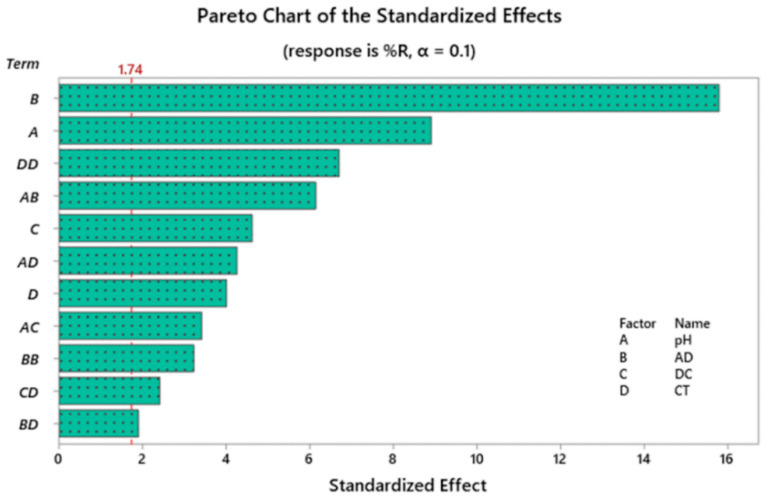
Pareto chart of standardized effects following response transformation.

In order to investigate the statistical significance of tested variables, Pareto chart of standardized effects, normal and half-normal probability plots alongside with analysis of variance (ANOVA) were implemented. Pareto chart (Figure 1) shows that AD (B) is the most statistically effective factor, followed by the effect of pH (A). It can be also observed that the CT is not that much effective compared to the other factors, however the squared interaction (CT × CT) was the third most influencing variable. The interaction of the CT × AD was the least effective factor on the %R of RB dye. Similar conclusions were obtained using the analysis of variance test (ANOVA) at 95.0 confidence interval (95.0 CI). ANOVA results are shown in Table 4. F-value is shown for every model term and is sufficiently large in case of statistically significant variables. As shown in the table as well, variables with a significance level (*p*-value) less than 0.05 are statistically significant, and the opposite is true. Table 4 also shows that lack-of-fit has a *p*-value of 0.633 (statistically not significant) inferring goodness-of-fit.

It is noteworthy to mention that response surface regression was performed versus blocks, pH, AD, DC as well as the CT employing Box-Cox transformation [37] where the transformation factor, λ = 0.75 and backward elimination of terms (α to remove = 0.1) was used, Equation (3):

(Transformed response) Y′= (Y_λ_ − 1)/λ (transformation factor) (3)

The outcome of the response surface regression is the following mathematical paradigm shown in Equation (4):

(4) $$\begin{array}{l} {\%R}^{0.75}=12.46-1.442\;\mathrm{pH}+1134\;\mathrm{AD}-0.450\;\mathrm{DC}-0.1495\;\mathrm{CT}-7465\;\mathrm{AD}\times \mathrm{AD}\\ \;+0.000810\;\mathrm{CT}\times \mathrm{CT}-42.21\;\mathrm{pH}\times \mathrm{AD}+0.0468\;\mathrm{pH}\times \mathrm{DC}\\ \;+0.00668\;\mathrm{pH}\times \mathrm{CT}-0.448\;\mathrm{AD}\times \mathrm{CT}-0.001133\;\mathrm{DC}\times \mathrm{CT}, \end{array}$$

Equation (4) shows that increasing the pH value would reduce the %R. Conversely, increasing the dose of RPAL would enhance the removal of RB. Model summary shows that the value of R^2^ was relatively high (R^2^ = 96.95%) and close to the value of R^2^—adjusted (R^2^ (adj) = 94.27%), indicating the linearity of the proposed model. The value of R^2^—predicted was also high (R^2^(pred) = 89.99%), implying that the proposed model is significantly capable of detecting new observations. This finding could be further confirmed by referring to Table 3 where both experimental and predicted values are revealed together with the difference between the experimental and actual values, relative to the actual values expressed as the relative error (RE). The shown error is relatively small reflecting a close match between observed and predicted responses.

### 2.4. Contour Plots of %R and Surface Optimization

**Figure 2:**
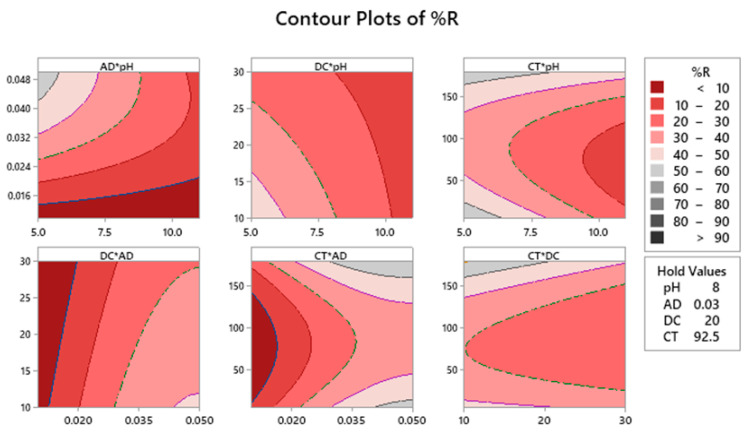
Response contour plots for the %R. Dark grey regions represent regions where maximum %R could be obtained using the factorial combination in each panel.

Figure 2 illustrates the two–dimensional (2D) plots for the measured fitted response surface. Each of the shown panels reveals the effect of two factors on %R. As shown in the attached legend, the dark red color implies a lower %R, while dark grey color means higher %R. Having the upper left panel as an example (AD × pH), having an AD of 0.042–0.048 g/50 mL and a pH level of 5.00–5.20, the %R is in the range of 50%–60%. Similar conclusions can be obtained from the rest of panels for each factorial combination. 

**Figure 3:**
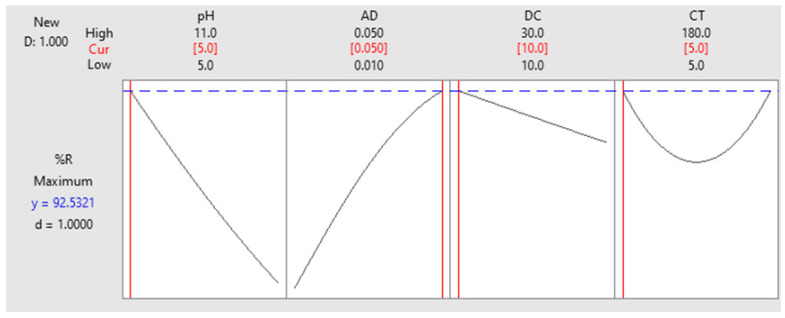
Optimization plot. A factorial combination of pH = 5.00 ± 0.20, AD = 0.050 g/50 mL, DC = 10 ppm and CT of 5 min. would achieve %R = 92.53%.

A typical strategy to deal with how a mixture of factorial settings satisfies the destinations that they were setup for is the use of “optimization plot”—a tool offered by Minitab to optimize the measured response. As shown in Figure 3, the objective was set to attain a 100% removal of RB, and the variable settings were fluctuated to achieve the objective. As shown, a blend of the tested variables at the level denoted as ‘Cur’ would produce a response value of 92.53%. The desirability value (*d*) was high enough, entailing the favorability of the mentioned blend. Figure 3 also shows that increasing the dose of RPAL enhances the adsorption process. This can be attributed to the increase in the number of adsorption sites available for the uptake of RB. The figure also shows that increasing the pH would decrease the %R, and similarly the DC. Impact of CT and as shown in the figure has a varying effect, where increasing the CT from 5 to 92.5 min. has resulted in reduced %R, while with increasing the time from 92.5 to 180 min., removal was improved. These findings are similar to the conclusions obtained from the mathematical paradigm described in Equation (4). Yet, explanation of these findings will be considered in lights of surface chemistry and nature of the dye throughout the next subsections.

### 2.5. Adsorbent Characterization 

#### 2.5.1. Thermogravimetric Analysis (TGA) 

**Figure 4:**
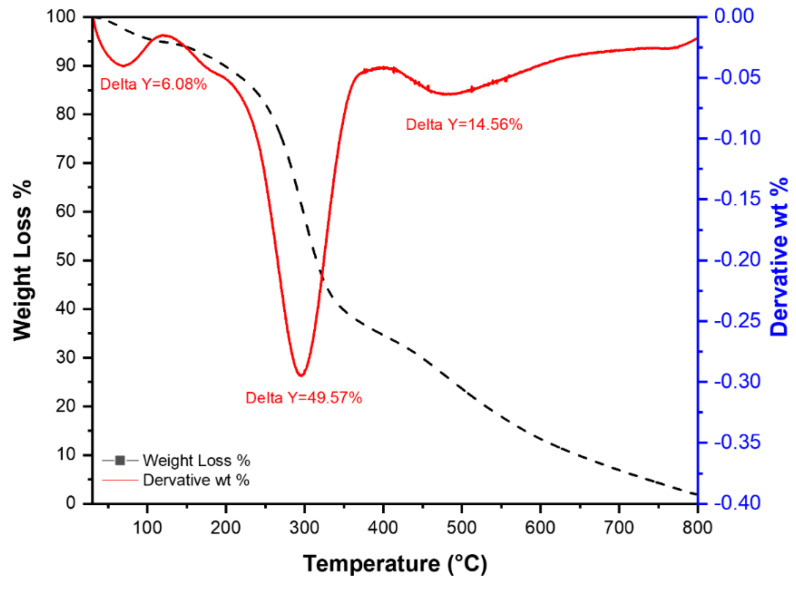
TGA graph of air—dried pineapple leaves (RPAL).

Thermogravimetric analysis of RPAL was done under N_2_ with a heating rate of 10 °C/min. The data represented in Figure 4 shows that the weight loss for the RPAL sample occurs over three steps as follows:

(1) First step: loss of adsorbed water molecules at a temperature range of 25–100 °C and represents 6.08% of the sample,
(2) In this step, >49% of the sample is decomposed between (200–500 °C) including the loss of crystalline water at ~200 °C and part of the organic matter, as represented by a major peak at 305 °C, which could be ascribed to the decomposition of the organic material in RPAL,
(3) The last step at ~525 °C where 14.46% of the RPAL sample was lost at this stage and it could be related to the carbonization of PAL.

As shown from the TGA findings, thermal treatment of the RPAL might have resulted in the evaporation of the small molecules such as H_2_O, CO, and CO_2_. Absence of these functionalities in the thermally treated samples would explain their diminished adsorption capabilities compared to the RPAL sample.

#### 2.5.2. Fourier Transform Infrared Spectroscopic Analysis (FT-IR)

**Figure 5:**
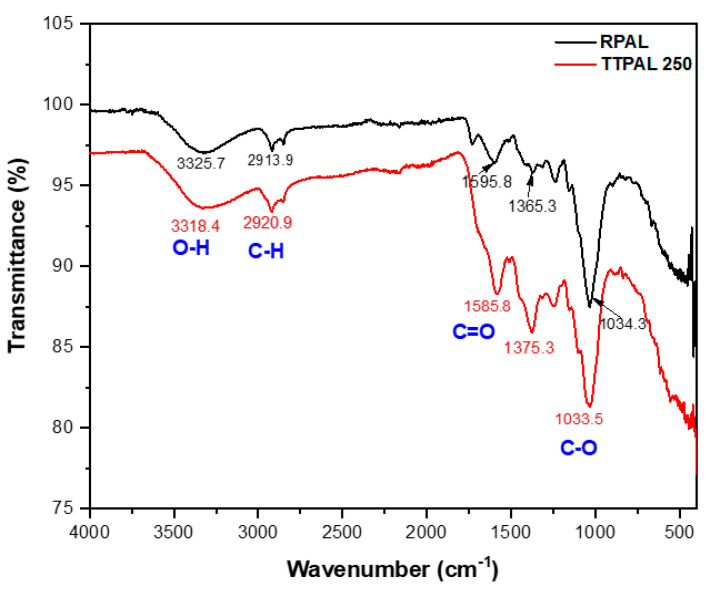
FT-IR spectra of RPAL and TTPAL250.

FT-IR spectra of RPAL and TTPAL250 are given in Figure 5. As previously indicated, PAL are mainly composed of lignocellulosic material [28,29,32]. The obtained spectra show the existence of almost the same peaks in the two samples but with lower intensity in the thermally treated one due to the decomposition of lignocellulosic material, a finding that explains the subordinate adsorption capability of the later compared to the former [33]. The obtained data show a broad absorption band centered at 3325 cm^−1^ for the RPAL and 3318.4 cm^−1^ for TTPAL250. This peak could be assigned to the hydrogen–bonded—OH vibration of the cellulosic structure of the RPAL. In addition, it could be attributed to N-H group which is confirmed later by the presence of a high concentration of nitrogen in both raw and thermally treated samples in the CHN analysis. The spectra also show the presence of the absorption band at 2913–2920 cm^−1^ in both samples, which could be ascribed to the C-H stretching of aliphatic—CH groups. The absorption bands at 1595–1585.8 cm^−1^ confirm the presence of bending N-H of amines. The two bands at 1365 and 1375 cm^−1^ can be assigned to bending—OH. The absorption band at 1034.3 cm^−1^ for the RPAL and 1033.5 cm^−1^ for TTPAL250 can be ascribed to the presence of C–O stretching. The FT-IR results confirm the presence of surface functional groups that should have played an important role in the adsorption of RB onto PAL.

By combining the FT-IR data together with the FCCCD analysis findings, it can be recognized that the pH has a substantial influence on RB sorption process. Measurements were made at three pH values 5.00, 8.00, and 11.00. These values were carefully selected, where RB had the same absorption maxima in the three solutions. Moreover, the color of RB disappeared at pH less than 4.00. As per the design analysis, biosorption of RB onto RPAL was maximum at the acidic side (pH = 5.00 ± 0.20) and further elevation in the pH has resulted in a diminutive removal, Figure 3. RB and as previously reported, is an anionic dye with a pKa value of 4.50 [38,39]. Therefore, at pH > pKa, RB will start to be ionized (deprotonated, negatively charged). On the other hand, the surface of RPAL at the acidic side and as per the FT-IR analysis might have some positively charged functionalities. The existence of negatively charged RB on the positively charged RPAL surface would encourage electrostatic interaction. Conversely, at pH = 11.00 surface of RPAL will be negatively charged, therefore, less interaction between RB and RPAL surface. Similar results for better sorption of RB in acidic media has been previously reported using different adsorbents such as Fe (III)–montmorillonite [16], chitosan–TiO_2_ nanocomposite [17], and bottom ash [40].

#### 2.5.3. Raman Analysis

**Figure 6:**
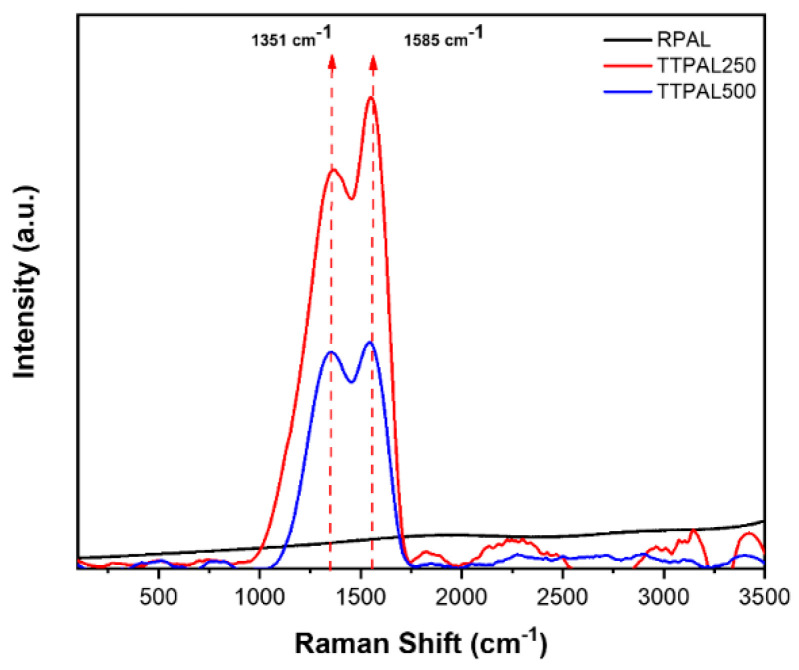
Raman spectra of the raw pineapple leaves (RPAL) and the thermally treated samples (TTPAL250 and TTPAL500).

Raman spectra of raw and thermally treated pineapples are shown in Figure 6. The obtained spectra show the absence of any peaks in the range between 1000 to 2000 cm^−1^ in the raw sample. This could be explicated taking in consideration that carbon in the raw sample exists in the form of organic matter. Contrariwise, the Raman spectra of the burnt samples (TTPAL250 and TTPAL500) show two peaks which could be ascribed to the D–and G–bands at approximately 1351 cm^−1^ (D–band) and 1585 cm^−1^ (G–band). It is imperative to mention that these two bands are characteristic peaks for carbon materials. In addition, the resulted D–, and G–bands pattern is close to the bands present in graphene oxide [41]. Besides, the D–band reflects the carbon lattice properties including defects and sizes, but the G–band shows the stretching of C-C in sp^2^ system [42]. Furthermore, the ration between intensity of D–band to G–band was calculated (I_D_/I_G_) and compared for the two thermally treated samples. Interestingly, the I_D_/I_G_ for TTPAL250 was 0.90 compared to 1.07 for TTPAL500. This finding confirms the fact that the number of defects has increased by increasing the burning temperature. Yet, it can be also observed that the burning process (carbonization) might have resulted in the elimination of some essential functional groups, which in turn might have an important role in the diminished removal efficiency of the TTPAL250 and TTPAL500 compared to RPAL sample.

#### 2.5.4. Scanning Electron Microscopy Analysis (SEM)

**Figure 7:**
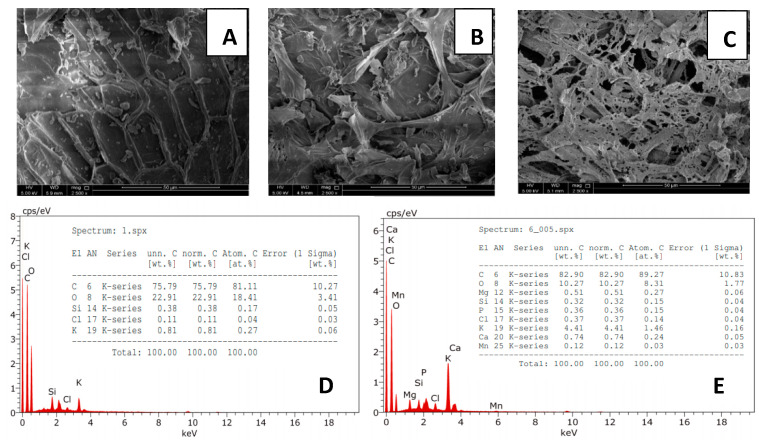
The upper panel is the SEM micrographs of RPAL (**A**), TTPAL250 (**B**), and TTPAL500 (**C**). The lower panel is the EDX analysis of RPAL (**D**), and TTPAL500 (**E**).

The surface structure of the raw and the thermally treated PAL was explored using the scanning electron microscope (SEM). The SEM micrographs presented in Figure 7 showed that the RPAL (Figure 7A) has plain surface without any pores and the same was also observed following the burning process at 250 °C (Figure 7B). On the other hand, the surface has completely changed after burning at 500 °C. Figure 7C shows the presence of high porous surface compared to the raw material, confirming the formation of carbonaceous material with advanced pore structure and the loss of organic matter after burning at 500 °C. These findings are in a good match with the obtained data by FT-IR and TGA analyses. Furthermore, EDX analysis shows the effect of the burning process on the concentration of carbon and oxygen. Results show that carbon content has increased from 75.79% in the RPAL to 82.90% in the burnt sample (Figure 7D,E). In addition, the oxygen content has decreased from 22.91% in the RPAL to 10.27% in RPAL500. This decrease might be attributed to the loss of water oxygen during the burning process, an issue that might have a negative impact on the removal efficiency of the thermally treated samples and as was confirmed by the FT-IR and Raman analyses.

#### 2.5.5. Carbon, Hydrogen, and Nitrogen Analysis (CHN)

Data shown in Table 5 represent a comparison between three samples RPAL, TTPAL250, and TTPAL500 in terms of the percentage Carbon, Hydrogen, and Nitrogen. The collected data show that the %C and %N has increased following the thermal treatment in contrast to the %H. These findings indicate that the burning process might cause the loss of hydrogen in crystalline and physical water in contrast to the carbon concentration, which has increased because of the conversion of the biomass into carbon during the burning process.

#### 2.5.6. Brunauer–Emmett–Teller (BET) Surface Area Analysis

**Figure 8:**
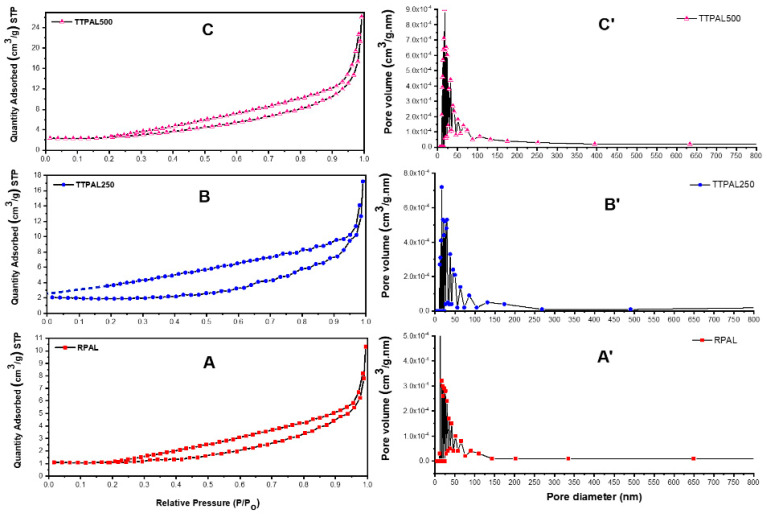
BET analysis of (**A**) RPAL, (**B**) TTPAL250, and (**C**) TTPAL500.

Table 6 shows the measured BET surface area and the total pore volume of the three adsorbents using N_2_ adsorption–desorption measurements. The obtained data show that the surface area of RPAL is 4.59 m^2^/g and this area has increased (almost doubled) following thermal treatment to 9.81 m^2^/g for TTPAL500 with no much difference between TTPAL250 and TTPAL500. On the other hand, the total pore volume has increased from 0.016 to 0.041 cm^3^/g for RPAL and TTPAL500, respectively. This increase in the pore volume is confirmed by the SEM micrographs. Conversely, the pore radius has decreased in the thermally treated samples compared to the raw one. These findings together with the FT-IR and Raman, and FCCCD analyses might explain the superiority of RPAL as adsorbent compared to the TTPAL250 and TTPAL500 samples, and confirm that the adsorption process is controlled by the chemical structure of the adsorbent surface, which in turn is affected by the adsorption conditions. Figure 8 displays that the three adsorbents show a type III adsorption isotherm with H3—hysteresis loop, indicating the unrestricted multilayer formation and that lateral interactions between the adsorbate molecules are stronger than the interactions between adsorbent and the adsorbate. The H3—hysteresis indicates the aggregation of plate–like particles to form slit–like pores in loose assemblies. Furthermore, it also shows the presence of two types of pores including mesopores (2–50 nm diameter) and macropores (>50 nm diameter, according to the IUPAC classification), in alignment with the analysis of SEM micrographs, Figure 7 [43].

### 2.6. Equilibrium and Kinetics Studies of the Adsorption of RB onto PAL

The data displayed in Table 2 prove that RPAL has higher adsorption efficiency compared to the thermally treated samples, hence, the equilibrium isotherms and kinetics studies were carried out using the RPAL sample. Important information, such as the maximum quantity adsorbed, the type of interaction (chemi—or physisorption) between the adsorbate and the adsorbent surface, are by and large obtained using adsorption isotherms. Kinetics studies, on the other hand, are used to find the different factors affecting the adsorption process including adsorption rate, type of the layer formed on the surface of the adsorbent (mono or multilayer), and the type of the adsorption mechanisms. The data given below will show the kinetics and adsorption isotherms of the adsorption of RB dye onto the RPAL sample.

#### 2.6.1. Equilibrium Isotherms

**Figure 9:**
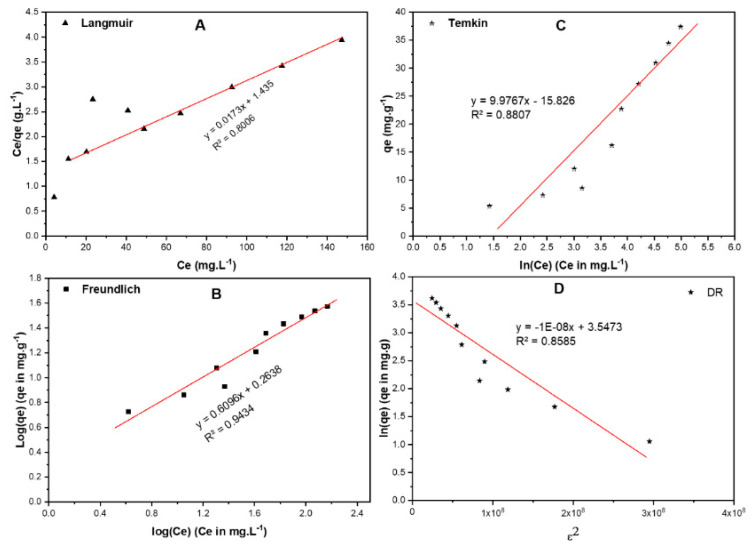
Adsorption isotherms of RB on RPAL including (**A**) Langmuir, (**B**) Freundlich, (**C**) Temkin, and (**D**) Dubinin–Radushkevich (DR).

The biosorption of RB dye onto the RPAL was studied using four isotherms: (1) Langmuir, (2) Freundlich, (3) Temkin, and (4) Dubinin–Radushkevich (DR) paradigms [44,45,46,47]. Single–layer homogeneous adsorption on the surface of the adsorbent was explained by Langmuir isotherm as shown in Figure 9A and Table 7. The Langmuir equation is shown below:

(5) $$q_{e}=\frac{q_{m}\;K_{L}\;C_{e}}{1-K_{L}\;C_{e}}$$

In Equation (5), *q_m_* and *K_L_* stand for the maximum adsorption capacity and the Langmuir equilibrium coefficient, respectively. Langmuir equation can be expressed using the following formula:

(6) $$R_{L}=\frac{1}{1-K_{L}\;C_{0}}$$

where *R_L_* and *C*_0_ represent the separation factor and the initial concentration (mg/L) respectively. The *R_L_* value reflects the feasibility of the sorption process. Therefore, if *R_L_* is higher than 1, the adsorption process is counted as unfavorable and if *R_L_* is equal to 1, the adsorption isotherm is linear. In cases where the *R_L_* value is in the range between 0 and 1, then the adsorption process is favorable, and it occurs spontaneously, while if *R_L_* is equal to 0, the adsorption is expressed as irreversible process [47]. Based on the obtained data for the current work, the *R_L_* value was found to be less than 1 and higher than 0, indicating that the biosorption of RB onto RPAL was spontaneous and the monolayer maximum adsorption capacity (*q_max_*) = 58.80 mg/g.

The heterogeneous adsorption is usually portrayed using the Freundlich isotherm described by the following equation:

(7) $$q_{e}=K_{F}C_{e}^{\frac{1}{n}}$$

where *C_e_* is the equilibrium concentration of RB (mg L^−1^); *q_e_* is the amount of RB adsorbed/unit mass (mg·g^−1^), while K_F_ (mole·g^−1^) (L·mole^−1^)^1/*n*^ and 1/*n*, are the Freundlich coefficients. This model presumes neither homogenous adsorption nor restricted level of biosorption. According to the obtained data shown in Figure 9B and Table 7, the highest R^2^ value (0.943)–among the four studied models—was obtained using the Freundlich isotherm, implying that this model holds for the RB—RPAL system. Freundlich coefficient 1/*n* = 0.609 and *n* = 1.642, signifying that the biosorption of RB is favorable where the value of 1/*n* < 1. This isotherm also designates that the adsorption might not be monolayer and that adsorption sites with higher affinity might be inhabited first. This finding also explains why the removal efficiency (%R) has decreased with increasing [RB].

The adsorbate–adsorbent interaction was also studied using the Temkin isotherm as shown in Figure 9C and Table 7. Temkin isotherm, however, cannot be used to explain the adsorption of RB onto RPAL since the R^2^ value = 0.881. The DR isotherm, (Figure 9D and Table 7) was used to detect the type of adsorption on a heterogeneous surface [47]. Based on the reported information on the relation between the free energy value and the adsorption mechanism, where if the free energy value is <8.0 kJ/mol, the adsorption process is physisorption while if the free energy is >8.0 kJ/mol then the adsorption process will be chemisorption. According to the data revealed in Table 7, the free energy for adsorption of RB onto RPAL is physisorption where the amount of free energy equals 7.07 kJ/mole. Yet, this type of isotherm might not be applicable in the current investigation where data did not show an excellent goodness–of–fit with R^2^ = 0.858. These findings together with the characterization outcomes show that and though free energy implies physisorption, occurrence of chemisorption cannot be ruled out [48].

#### 2.6.2. Biosorption Kinetics

**Figure 10:**
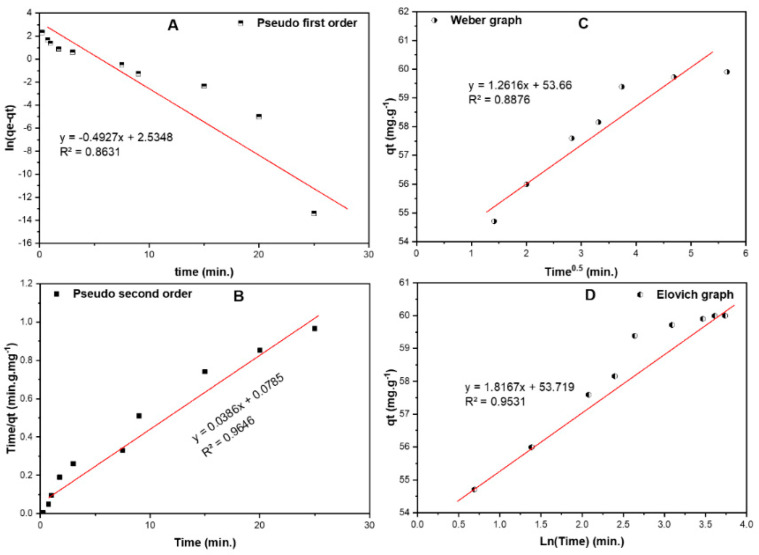
Kinetic models for the adsorption of RB on RPAL including (**A**). Pseudo first order, (**B**). Pseudo second order, (**C**). Elovich and (**D**). intra–particle diffusion (Weber–Morris) curves.

In this study, four models were tested; pseudo–first order (PFO), pseudo–second order (PSO), Elovich and Weber–Morris (W–M) to explain the kinetics of the adsorption process of RB onto RPAL. The data shown in Figure 10A,B represent the plots of [ln(*q_e_*–q_t_) vs. time] and [time/*q_t_* vs. time] for the two tested kinetic models; PFO and PSO, respectively. Other parameters together with their values are listed in Table 8. By comparing the linearity and the calculated adsorption capacity at equilibrium for these two models, it can be detected that the PSO model is more applicable in explaining the adsorption of RB onto RPAL [49,50,51]. Therefore, the reaction of RB with RPAL can be expressed as:

(8) $$RB+RPAL\;\overset{k}{\to }\;\left\{RB-RPAL\right\}$$

Therefore, the rate of the reaction can be expressed as: *k*[RB][RPAL], implying that the adsorption rate depends mainly on both RB and RPAL concentrations. Weber–Morris intra–particle diffusion model, Figure 10C, indicates that the diffusion rate is very fast with the value of K_1_ = 1.262. The mechanism of adsorption process using this model involves the formation of a layer of RB around the particles of RPAL, which will prevent any penetration of more RB and form a boundary layer (53.66 mg/g). This value is close to the *q_max_* obtained from the Langmuir isotherm. Finally, the Elovich model, Figure 10D, shows a low R^2^ value (0.953) compared to PSO model. This model shows that the initial adsorption rate (α = 3.79 × 10^12^ mg·g^−1^·min^−1^) is higher than the desorption rate (β = 1.817 g·mg^−1^). Therefore, the adsorption of RB onto RPAL involves a second–order uptake rate vs. the existing surface sites.

## 3. Materials and Methods 

### 3.1. Materials and Reagents

The chemicals used were of the analytical grade and were used as acquired with no additional purification. Sodium hydroxide, sodium tetraborate–10–hydrate and hydrochloric acid were purchased from Sigma–Aldrich (Eschenstrasse, Taufkirchen, Germany). Rose bengal (RB) was a product of BDH Laboratory Supplies (Poole, UK). Values of pH were adjusted as previously mentioned [10]. Pure water was used for diluting the RB dye solutions to 1000 ppm. Pineapple leaves (PAL) were used after drying as will be described in their method of preparation.

### 3.2. Instrumentation and Software

A Jenway pH meter was used for the preparation of different pH dye solution. An ST8 Benchtop Centrifuge (Thermo Scientific, Waltham, MA, USA) was used for separating the components of each sample mixture. The absorbance was measured using an UV–Vis spectrophotometer (Agilent DAD, Agilent, Santa Clara, CA, USA). The surface morphology of the prepared pineapple leaves was identified using a scanning electron microscope (SEM– Quanta 200, Thermo Scientific, Waltham, MA, USA) and energy–dispersive X–ray spectroscopy (EDX, Thermo Scientific, Waltham, MA, USA). Fourier transform infrared radiation (FT-IR, Bruker Alpha, MA, USA) was used to determine the functional groups on the surface of pineapple leaf. The Raman spectrum was recorded in the range from 50–3500 cm^−1^ using a Raman microscope (DXR Raman Microscope, Thermo Scientific, Waltham, MA, USA), with a laser beam at 532 nm as excitation source. Furthermore, a thermal gravimetric analyzer (TGA400, PerkinElmer, Waltham, MA, USA was utilized to inspect the thermal stability of the pineapple leaf. Finally, Minitab^®^19 software (Minitab Inc., Chicago, IL, USA) was used to construct the face–centered central composite design (FCCCD).

### 3.3. Face—Centered Central Composite Design (FCCCD)

The design of experiment chosen to conduct the current study is FCCCD. The percentage removal (%R) as a single response was optimized as a function for four independent variables, pH, DC, AD, and CT (Table 3). The design matrix involved conducting 30 basic runs in one replicate over two blocks with α = one. Design points involved 16 cube points, eight axial points, and total of six center points. The full design matrix as shown in Table 3.

### 3.4. Preparation of RB

Ultra–pure water was artificially contaminated with RB dye to have a stock solution of 1000 ppm. Serial dilutions of the RB solution were prepared by adjusting the desired pH value using the previously prepared pH adjusting solutions. Three calibration curves were prepared, therefore, at three pH values, Table 3, and measured at 548 nm.

### 3.5. Adsorbent Preparation

#### 3.5.1. Air–Dried Raw Pineapple Leaves (RPAL)

Pineapples were purchased from a local market in Doha–Qatar. The leaves of the pineapple were separated from the bottom of the pineapple fruit using a metal blade. The crown base was detached, then the pineapple leaves were cut into small pieces approximately 1 × 1 cm. These pieces were rinsed with tap water followed by distilled water to remove any impurities or pollutants present on their surface. The cut leaves were then dried and exposed to the sunlight directly for three consecutive days until they are completely dry. Dry leaves were allotted as three portions. The first portion was further dried in air and labeled as raw pineapple leaves (RPAL).

#### 3.5.2. Thermal Treatment of Pineapple Leaves 

Portions 2 and 3 were activated in the oven at 250 °C and 500 °C for 1 h, and labeled as, thermally treated pineapple leaves; (TTPAL250), and (TTPAL500), respectively. The three portions and after the previous treatment were chopped well with electrical grinder until it becomes fine powder.

### 3.6. Evaluation of the Adsorption Perfomance of the Prepared Adsorbents 

Two batches of 15 mL centrifuge tubes were prepared. The first set was the sample and the second set was for the blanks. In each tube for both sets, 30–150 mg of RPAL was added. The pH value of the RB solutions was adjusted to the desired figure (Table 3). Next, the two sets of samples and blanks were centrifuged at 4200 rpm for the time specified in Table 3 to facilitate obtaining the supernatant. UV–Vis spectrophotometer was used to measure the absorbance of the supernatant.

## 4. Conclusions

The present work has emphasized that economic PAL adsorbents could be efficiently used for the adsorption of rose bengal (RB) from wastewater. Three types of adsorbents were developed for that purpose, raw (RPAL) and thermally treated PAL at 250 °C and 500 °C. Results showed that RPAL is more efficient for the removal of RB. A smart and ecofriendly platform has been proposed to engineer the removal process. In this context, a response surface methodological approach (face–centered central composite design, FCCCD) was used to optimize the variables influencing the adsorption process. The response (%R) was measured as a function of four factors (pH, AD, DC, and CT). As per the response surface regression model, increasing the dose of RPAL improves the adsorption of the dye, in contrast to pH and DC. FT-IR and Raman spectra were used to examine the prepared adsorbents. FT-IR data showed the presence of–OH, N–H, C–H, and C–O function groups in RPAL as well as in the thermally treated sample but with a lower intensity. Raman spectra showed the formation of carbonaceous material after the burning process as confirmed by the presence of D– and G–bands. The equilibrium studies revealed that the biosorption of RB on RPAL could be represented by the Freundlich isotherm. The maximum monolayer adsorption capacity was 58.80 mg/g as determined by the Langmuir isotherm. Furthermore, the adsorption of RB onto RPAL is physisorption with free energy equals 7.07 kJ/mol as calculated by the Dubinin–Radushkevich (DR) isotherm. However, and considering the SEM and BET analyses together with the FT-IR findings, occurrence of chemisorption cannot be ruled out. The kinetic studies showed that the adsorption process was a second–order reaction and adsorption rate depends mainly on both RB and RPAL concentrations.

## Figures and Tables

**Table 1:** Evaluation of the performance of pineapple leaf (PAL) processed in current work compared with other studies used PLP as adsorbent for removal different adsorbates.

Absorbent	Modification Method	Analytical Approach Used	Surface Area (m^2^/g)	Adsorbate	Adsorption Capacity (mg/g)	%Removal	References
**Raw pineapple leaves (RPAL)**	Please check the experimental part of this paper.	FCCCD	4.79	Rose Bengal	58.8	92.53%	Current work
**Pineapple leaf powder (PLP)**	Leaves were washed, dried at 80 °C for 24 h, at pressure 70 kPa, pulverized, and grinded to fine PLP to be used.	Single variate analysis	ND *	Cu (II)	9.28	90%	[29]
**NaOH-treated pineapple waste**	Leaves were washed several times, dried in oven at 105 °C for 24 h, grinded and screened by 60 mesh sieves to use.	Single variate analysis (Batch experiments)	ND *	Pb (II) and Cd (II)	ND *	<95%	[30]
**Surface modified pineapple crown leaves (PCL)**	Leaves were washed, dried at 70 °C for 48 h, pretreated by isopropyl alcohol and NaOH to produce (P)PCL, modified by acetic acid and hydrogen peroxide to produce (M)PCL.	Single variate analysis	(P)PCL: 32.90	Cr (VI) and Cr (III)	Cr(VI) on (M)PCL: 3.91 Cr(VI) on (P)PCL: 2.69 Cr(III) on (M)PCL: 2.54 Cr(III) on (P)PCL: 1.82	ND *	[31]
**Pineapple leaf powder (PLP)**	Leaves were washed several times, dried in oven at 80 °C for 48 h, grinded into powder for further use.	Single variate analysis	5.24	Methylene Blue	ND *	<95%	[32]
**Pineapple crown activated carbon and ZnCl_2_**	Leave were washed with distilled water, dried at 110 °C, chopped into small pieces, and mixed at ratio 1:1 with zinc chloride.	Single variate analysis (Batch experiments)	914.7	Methylene Blue	288.34	* ND	[33]
**Pineapple leaf powder**	Leaves were washed several times, dried in oven at 105 °C for 24 h, grinded and sieved to fine powder to use.	Single variate analysis	ND *	Remazol Brilliant Blue R	9.58	<90%	[34]
**Pineapple leaf powder**	Leaves were washed with distilled water, dried in oven at 105 °C, crushed, and sieved.	Single variate analysis (Batch experiments)	ND *	Methylene Blue	78.13	* ND	[35]

* ND: Not Determined.

**Table 2:** Performance of PAL—based adsorbents in terms of %R and *q_e_*. Testing adsorption performance was conducted using a variable blend of pH = 7.00 ± 0.20, DC = 50 ppm, AD = 50 mg/15 mL, CT = 30 min. The responses shown were calculated using Equations (1) and (2).

Adsorbent Type	Percentage Removal (%R)	Adsorption Capacity (*q_e_*, mg/g)
RPAL	42.96	6.44
TTPAL250	18.99	2.85
TTPAL500	18.53	2.78

**Table 3:** Independent factors and their levels together with the observed and predicted dependent variable and the FCCCD matrix.

Factors					Low Level		Medium Level		High Level	
pH					5		8		11	
Adsorbent Dose (AD, B, g/50 mL)					0.01		0.03		0.05	
Dye Concentration (DC, C, ppm)					10		20		30	
Contact Time (CT, D, min)					5		92.5		180	
**Experimental Runs, Observed and Predicted Responses**										
Expt No	Blk *	pH	AD	DC	CT	%R Obs. **		%R Pred. **		RE ***
01	1	5(−)	0.03(0)	20(0)	92.5(0)	38.19		36.20		0.05
02	1	8(0)	0.01(−)	20(0)	92.5(0)	1.43		2.83		0.49
03	1	8(0)	0.05(+)	20(0)	92.5(0)	47.06		35.19		0.34
04	1	8(0)	0.03(0)	20(0)	92.5(0)	33.00		25.36		0.30
05	1	8(0)	0.03(0)	20(0)	5(−)	38.19		40.15		0.05
06	1	8(0)	0.03(0)	10(−)	92.5(0)	34.72		30.87		0.12
07	1	11(+)	0.03(0)	20(0)	92.5(0)	23.41		15.59		0.50
08	1	8(0)	0.03(0)	30(+)	92.5(0)	28.15		20.14		0.40
09	1	8(0)	0.03(0)	20(0)	180(+)	62.73		50.91		0.23
10	1	8(0)	0.03(0)	20(0)	92.5(0)	19.32		25.36		0.24
11	1	5(−)	0.01(−)	10(−)	5(−)	25.46		24.28		0.05
12	1	5(−)	0.01(−)	30(+)	5(−)	7.84		12.17		0.36
13	1	11(+)	0.05(+)	10(−)	5(−)	21.14		26.06		0.19
14	1	8(0)	0.03(0)	20(0)	92.5(0)	19.24		25.36		0.24
15	1	5(−)	0.01(−)	30(+)	180(+)	10.40		10.18		0.02
16	2	5(−)	0.05(+)	10(−)	5(−)	86.64		92.53		0.06
17	2	11(+)	0.01(−)	10(−)	180(+)	26.86		30.36		0.11
18	2	5(−)	0.05(+)	10(−)	180(+)	88.40		92.60		0.04
19	2	5(−)	0.01(−)	10(−)	180(+)	27.07		34.02		0.20
20	2	11(+)	0.01(−)	10(−)	5(−)	1.88		3.88		0.51
21	2	5(−)	0.05(+)	30(+)	5(−)	71.46		74.71		0.04
22	2	8(0)	0.03(0)	20(0)	92.5(0)	23.71		25.36		0.06
23	2	11(+)	0.01(−)	30(+)	180(+)	17.04		22.04		0.23
24	2	8(0)	0.03(0)	20(0)	92.5(0)	20.07		25.36		0.21
25	2	11(+)	0.01(−)	30(+)	5(−)	5.14		6.29		0.18
26	2	5(−)	0.05(+)	30(+)	180(+)	50.70		59.65		0.15
27	2	8(0)	0.03(0)	20(0)	92.5(0)	23.41		25.36		0.08
28	2	11(+)	0.05(+)	10(−)	180(+)	44.26		49.17		0.10
29	2	11(+)	0.05(+)	30(+)	180(+)	31.96		39.67		0.19
30	2	11(+)	0.05(+)	30(+)	5(−)	25.45		29.75		0.14

* Blk: Block; ** Obs: observed readings; ** Pred.: predicted readings; *** RE = Relative error = */* (*Measured value* − *Actual value*)/Actual value */*.

**Table 4:** Analysis of variance (ANOVA) for the transformed response.

	DF *	Adj SS	Adj MS	F-Value	*p*-Value
Model	12	1329.53	110.794	40.79	0
Blocks	1	27.58	27.583	10.15	0.005
Linear	4	993.74	248.436	91.45	0
pH	1	215.12	215.12	79.19	0
AD	1	677.08	677.082	249.24	0
DC	1	58.02	58.024	21.36	0
CT	1	43.52	43.518	16.02	0.001
2–Way Interactions	5	209.07	41.813	15.39	0
pH × AD	1	102.61	102.609	37.77	0
pH × DC	1	31.67	31.665	11.66	0.003
pH × CT	1	49.25	49.254	18.13	0.001
AD × CT	1	9.81	9.814	3.61	0.074
DC × CT	1	15.72	15.724	5.79	0.028
Squared Interactions	2	123.74	61.872	22.78	0
AD × AD	1	28.23	28.234	10.39	0.005
CT × CT	1	121.83	121.825	44.85	0
Error	17	46.18	2.717	0.85	0.633
Lack–of–Fit	13	33.92	2.609		
Pure Error	4	12.27	3.066		
Total	29	1375.71			

* DF is degrees of freedom SS is sum of squares and MS is mean of squares.

**Table 5:** CHN Elemental analysis of the prepared adsorbents.

Adsorbent	%C	%H	%N
RPAL	39.555	4.991	2.447
TTPAL250	52.140	4.942	3.117
TTPAL500	50.353	2.529	2.438

**Table 6:** Brunauer–Emmett–Teller (BET) analysis of RAPL and thermally treated samples.

Parameters	RPAL	TTPAL250	TTPAL500
Langmuir SA (m^2^/g)	4.59	8.43	9.81
Total pore volume (cm^3^/g)	0.016081	0.02674	0.040636
Average pore radius (°A)	105.5	81.4	96

**Table 7:** General and linearized equation of Langmuir, Freundlich, Temkin and Dubinin–Radushkevich isotherms, beside their parameters for the adsorption of RB on ADPP.

Isotherm	Equations (Generalized/Linearized Forms)	Parameters	Value
Langmuir	$q_{e}=\frac{q_{m}\;K_{L}\;C_{e}}{1-K_{L}\;C_{e}}$	$q_{m}$ (mg/g)	58.80
		$K_{L}$ (L·mole^−1^)	0.012
	$\frac{C_{e}}{q_{e}}=\frac{1}{q_{m}\;K_{L}}+\frac{C_{e}}{q_{m}}$		
		R^2^	0.801
Freundlich	$q_{e}=K_{F}C_{e}^{\frac{1}{n}}$	1n	0.609
		$K_{F}$ (mole/g) (L/mole)^1/n^	1.835
	$\log\left(q_{e}\right)=\log\left(K_{F}\right)+\left(\frac{1}{n}\right)\log\left(C_{e}\right)$		
		R^2^	0.943
Temkin	$q_{e}=\frac{RT}{b_{T}}\;\ln\left(A_{T}\;C_{e}\right)$	$b_{T}$ (J/mole)	248.4
		$A_{T}$ (L/mole)	0.205
	$q_{e}=\frac{RT}{b_{T}}\ln\left(A_{T}\right)+\frac{RT}{b_{T}}\ln\left(C_{e}\right)$		
		R^2^	0.881
DR	$\ln(q_{e})=\ln\left(q_{m}\right)-\beta \epsilon^{2}$	β	1 × 10^−8^
	$\epsilon =RT\left(1+\frac{1}{C_{e}}\right)$	E (kJ/mole)	7.07
		$q_{s}$ (mg·g)	34.72
	E=12β	R^2^	0.858

**Table 8:** The kinetics study results corresponding to Figure 10.

Model	Parameter	Value
Pseudo–first order (PFO) $\ln\left(q_{e}-q_{t}\right)=\ln\left(q_{e}\right)-k_{1}t$	K_1_ (min^−1^)	0.493
	*q_e_* (mg/g)	12.61
	R^2^	0.863
Pseudo–second order (PSO) $\frac{t}{q_{e}}=\frac{1}{k_{2}q_{e}^{2}}+\frac{1}{q_{e}}$t *where* K_2_ *is rate constant* (*g·mg*^−1^·*min*^−1^)	K_2_ (g·mg^−1^·min^−1^)	0.019
	*q_e_* (mg/g)	25.91
	R^2^	0.965
Elovich model $q_{t}=\beta \ln\left(\alpha \beta \right)+\beta \ln\left(t\right)$ *where q_t_ is adsorbed quantity at time* t, α *is initial sorption concentration rate* (*mg·g^−1^·min^−1^), and β is desorption constant (g/mg).*	A	3.79 × 10^12^
	Β	1.817
	R^2^	0.953
Weber–Morris intra–particle diffusion model $q_{t}=K_{I}t^{0.5}+C$ *where* K_I_ *is intra–particle diffusion rate constant (mg·g^−1^·min^−0.5^), and* C *is boundary thickness effect*.	K_I_	1.262
	C	53.66
	R^2^	0.888
